# Outcomes of Bronchial Artery Embolization for Life-Threatening Hemoptysis in Patients with Chronic Pulmonary Aspergillosis

**DOI:** 10.1371/journal.pone.0168373

**Published:** 2016-12-22

**Authors:** Beomsu Shin, Won-Jung Koh, Sung Wook Shin, Byeong-Ho Jeong, Hye Yun Park, Gee Young Suh, Kyeongman Jeon

**Affiliations:** 1 Division of Pulmonary and Critical Care Medicine, Department of Medicine, Samsung Medical Centre, Sungkyunkwan University School of Medicine, Seoul, Republic of Korea; 2 Department of Radiology, Samsung Medical Centre, Sungkyunkwan University School of Medicine, Seoul, Republic of Korea; 3 Department of Critical Care Medicine, Samsung Medical Centre, Sungkyunkwan University School of Medicine, Seoul, Republic of Korea; Lee Kong Chian School of Medicine, SINGAPORE

## Abstract

**Background:**

Bronchial artery embolization (BAE) is an important treatment option for short-term control of hemoptysis in patients with simple aspergilloma (SA). However, there are no data on the outcomes of BAE in patients with chronic pulmonary aspergillosis (CPA). In this study, the clinical characteristics and outcomes of BAE were investigated and compared in patients with CPA and SA.

**Methods:**

We retrospectively analyzed the clinical data of 64 patients (55 [86%] with CPA and 9 [14%] with SA) who underwent BAE for life-threatening hemoptysis. The clinical characteristics and outcomes of BAE in CPA patients were compared to those of patients with SA.

**Results:**

The most common angiographic abnormality was hypervascularity (n = 60, 94%), followed by contrast extravasation (n = 50, 78%) and systemic-pulmonary shunt (n = 48, 75%), with similar incidence rates in both groups. Immediate success was achieved in 41 (64%) BAE procedures, but it was incomplete in 23 (36%) cases due to difficulty with the approach and/or overuse of contrast medium. Clinical failure of BAE was observed in only one (2%) patient. Complications following BAE were observed in four (6%) patients. Recurrence of hemoptysis was seen in a total of 33 patients (52%) within a median of 2.0 (0.3–10.0) months, and repeat BAE was performed in 25 (76%) of these cases. In comparing the outcomes of patients with CPA and SA, there were no differences in the rates of success of initial BAE, incomplete embolization, or clinical failure in the two groups. However, recurrence of hemoptysis tended to be higher in patients with CPA (55%) than in those with SA (33%). In addition, antifungal medications following BAE were more commonly prescribed in the CPA group (56%) compared to the SA group (0%).

**Conclusions:**

BAE was a safe and effective procedure for the management of life-threatening hemoptysis in patients with CPA. However, recurrence of hemoptysis was common, especially in patients with CPA. Therefore, definitive treatment for CPA following successful BAE should be considered to ensure the long-term success of the embolization in these patients.

## Introduction

The chronic forms of pulmonary aspergillosis refers to a spectrum of diseases, from simple aspergilloma (SA) to progressive cavitary disease,[1–4] a progressive pulmonary disorder that causes significant respiratory and systemic symptoms.[5] SA can exist for years without causing symptoms, although a minority of patients will experience hemoptysis.[6] In contrast to SA, progressive diseases such as chronic cavitary pulmonary aspergillosis, chronic necrotizing pulmonary aspergillosis, and chronic fibrosing pulmonary aspergillosis have high morbidity rates and are often complicated by subacute or massive hemoptysis.[1–6] SA, chronic cavitary pulmonary aspergillosis, chronic necrotizing pulmonary aspergillosis, and chronic fibrosing pulmonary aspergillosis are now collectively referred to chronic pulmonary aspergillosis (CPA).[7–14]

CPA can be complicated by life-threatening hemoptysis. Bleeding is usually occurs from systemic arteries supplying to the lungs, including bronchial, intercostal, subclavian, or internal mammary arteries.[15–17] Therefore, bronchial artery embolization (BAE) is an important treatment option for short-term control of hemoptysis significant enough to threaten clinical stability in patients with pulmonary aspergillosis.[1–3] Major complications of BAE are rare, and the immediate clinical success rate defined as hemorrhage cessation ranges from 85% to 100%, although recurrence of hemoptysis occurs in 10% to 33% of patients.[17] However, the outcomes of BAE in patients with CPA have never been studied, and there are limited data on BAE outcomes in patients with pulmonary aspergilloma.[18–21] Therefore, we investigated the clinical characteristics and outcomes of BAE in CPA patients with life-threatening hemoptysis compared to those of patients with SA.

## Methods

Data were collected from all patients with a clinical and radiological suspicion of pulmonary aspergillosis who underwent BAE for life-threatening hemoptysis at Samsung Medical Center (a 1,979-bed, university-affiliated, tertiary referral hospital in Seoul, South Korea) between January 2005 and January 2015. During the study period, a total of 530 patients underwent BAE for life-threatening hemoptysis. Of these patients, 92 patients were suspected to have pulmonary aspergillosis based on clinical and radiological findings. The medical records of these patients were reviewed, and a retrospective analysis was conducted. All patients with documented life-threatening hemoptysis preceding the BAE whose outcome could be ascertained for at least 6 months after BAE were included in the study. To qualify as ‘life-threatening’ hemoptysis, at least one of the following three criteria had to be documented in the patients’ case notes: 1) blood loss >200 ml/hour; 2) cumulative blood loss >600 ml/24 hours; or 3) respiratory compromise necessitating intubation and mechanical ventilation.[21]

The institutional review board of the Samsung Medical Center approved this study and waived the requirement for informed consent as we used only de-identified data collected as part of clinical practice.

### Diagnosis of CPA and SA

Diagnoses of CPA and SA were based on clinical, radiologic, microbiologic, and histopathologic findings and were thoroughly reviewed by three of the authors (B.S., W-J.K., and K.J.). Differences in observed findings were resolved by consensus. A diagnosis of CPA was considered accurate when it was associated with the following: (1) compatible chronic pulmonary or systemic symptoms, including at least weight loss, productive cough, or hemoptysis and elevated levels of inflammatory markers (C-reactive protein or erythrocyte sedimentation rate); (2) compatible chest radiological findings, including cavitary pulmonary lesion with or without evidence of paracavitary infiltration, new cavity formation, or expansion of cavity size over time; and (3) a positive serum *Aspergillus* precipitin test or isolation of *Aspergillus* species from a respiratory sample (i.e., sputum, transtracheal aspirate, or bronchial aspiration fluid).[3,8,11,12] *Aspergillus*-precipitating antibody tests were performed using an *Aspergillus fumigatus* IgG ELISA kit (IBL International, Hamburg, Germany). Diagnostic criteria for SA were presence of a fungal ball in a single pulmonary cavity with serological or microbiological evidence implicating *Aspergillus* spp. and no radiological progression over at least 3 months of observation in a non-immunocompromised patient exhibiting no or mild symptoms.[3]

### Embolization procedure and outcomes

The BAE procedure was performed by interventional radiologists. The choice of embolization method and materials was made by each individual physician. A catheter was introduced into the right femoral artery using a 5-French guiding catheter. Agents used for embolization included coils, Gelfoam, polyvinyl alcohol, or a combination of these materials. Immediate success of the BAE was defined as significantly reduced blood flow on angiography and no expectoration of fresh blood,[22] whereas failure was indicated by continued or recurrent hemoptysis within 24 hours after the first BAE. Recurrence was defined as expectoration of fresh blood 24 hours or more after the first BAE.[23]

### Data collection

The following data were collected from the electronic medical records: demographic data, comorbidities, respiratory or systemic symptoms, laboratory measurements, radiological findings including computed tomography (CT), and additional treatments for pulmonary aspergillosis. Angiographic and embolization parameters were extracted from the radiological reports. Finally, we documented the outcomes of patients including recurrence of hemoptysis, additional treatments for recurrence of hemoptysis due to pulmonary aspergillosis, and mortality.

### Statistical analysis

The data are presented as median and interquartile range (IQR) for continuous variables and as number and percentage for categorical variables. The data were compared using the Mann-Whitney *U* test or Kruskal-Wallis test for continuous variables and Pearson’s χ^2^ test or Fisher’s exact test for categorical variables. The Kaplan-Meier method was used to estimate the cumulative rates of recurrence following BAE, which were subsequently compared using the log-rank test. All the tests were two-tailed, and a *P*-value < 0.05 was considered significant. The data were analyzed using PASW Statistics 22 (SPSS Inc., Chicago, IL).

## Results

During the study period, a total of 92 patients suspected to have pulmonary aspergillosis underwent BAE for life-threatening hemoptysis. Of these patients, 64 (70%) patients with pulmonary aspergillosis (55 [86%] with CPA and 9 [14%] with SA) who underwent BAE for life-threatening hemoptysis were identified based on the eligibility criteria described above and included in the final analysis. The clinical characteristics of these patients are presented in Table 1. Most patients had underlying lung disease, such as previous tuberculosis (n = 49, 77%), bronchiectasis (n = 49, 77%), chronic obstructive pulmonary disease (n = 14, 22%), or non-tuberculous mycobacterial lung disease (n = 14, 22%). All patients presented with at least one of the following chest CT findings: cavitary lesion (n = 61, 95%), paracavitary infiltration (n = 49, 77%), mycetoma (n = 45, 70%), or consolidation (n = 33, 52%).

**Table 1:** Comparison of characteristics between patients with chronic pulmonary aspergillosis (CPA) and simple aspergilloma (SA) underwent bronchial artery embolization for life-threatening hemoptysis.

			All patients (N = 64)	CPA (n = 55)	SA (n = 9)	*P* value
Age, years			59 (50–68)	60 (51–68)	52 (50–66)	0.378
Gender, male			44 (69)	40 (73)	4 (44)	0.124
Body mass index, kg/m^2^			20.0 (15.9–23.5)	19.1 (15.6–23.0)	23.0 (19.4–25.0)	0.095
Comorbidities*						
	Underlying lung disease					
		Previous history of tuberculosis	49 (77)	43 (78)	6 (67)	0.427
		Bronchiectasis	49 (77)	44 (80)	5 (56)	0.196
		Chronic obstructive lung disease	14 (22)	13 (24)	1 (11)	0.670
		Nontuberculous mycobacterial lung disease	14 (22)	14 (26)	0	0.187
		Previous history of thoracic malignancy	5 (8)	5 (9)	0	> 0.999
		Interstitial lung disease	4 (6)	4 (7)	0	> 0.999
	Other comorbidities					
		Diabetes	13 (20)	10 (18)	3 (33)	0.372
		Chronic heart disease	9 (14)	8 (15)	1 (11)	> 0.999
		Chronic liver disease	5 (8)	5 (9)	0	> 0.999
		Previous history of extrathoracic malignancy	4 (6)	4 (7)	0	> 0.999
Chest computed tomographic findings*						
	Cavity		61 (95)	53 (96)	8 (89)	0.370
	Paracavitary infiltration		49 (77)	49 (89)	0	< 0.001
	Mycetoma		45 (70)	37 (67)	8 (89)	0.260
	Consolidation		33 (52)	29 (53)	4 (44)	0.729
	Bilateral involvement		5 (8)	5 (9)	0	> 0.999
Laboratory findings						
	White blood cells/μl		9,005 (7,518–11,710)	9,200 (7,920–11,940)	7,430 (5,325–9,630)	0.027
	Erythrocyte sedimentation rate, mm/hr		76 (42–113)	81 (53–116)	27 (20–44)	0.005
	C-reactive protein, mg/dl		2.57 (0.66–5.35)	2.62 (1.11–5.75)	0.24 (0.06–2.25)	0.004
	Albumin, g/dl		3.8 (3.5–4.2)	3.7 (3.4–4.1)	4.0 (3.8–4.6)	0.046
Microbiological tests*						
	Positive serum *Aspergillus* precipitin antibody test		50 (78)	48 (87)	2 (22)	< 0.001
	*Aspergillus* culture		38 (59)	30 (55)	8 (89)	0.071
Antifungal medications before embolization			18 (28)	18 (33)	0	0.052

* Cases are duplicated.

The data are presented as median (interquartile range) or number (%).

Compared with SA patients, the CPA patients were more likely to be male and to have low body mass index (Table 1). Inflammatory markers including leukocytosis, erythrocyte sedimentation rate, and C-reactive protein were higher in patients with CPA. In addition, cavity and paracavitary infiltration on chest CT scan were more commonly observed in patients with CPA than in patients with SA. However, there was no difference in comorbidities between the two groups. Eighteen (33%) patients with CPA were on antifungal treatment, while none of the SA patients had received this treatment before the BAE.

Angiographic findings and outcomes of BAE are shown in Table 2. Abnormalities of bronchial or non-bronchial arteries were found in all patients. The most common abnormality was hypervascularity in 60 (94%) patients, followed by contrast extravasation in 50 (78%), systemic-pulmonary shunt in 48 (75%), and neovascularization in 46 (72%) patients. Interestingly, the two groups did not demonstrate any differences in the types of vascular abnormalities. The bronchial arteries were the most frequently embolized vessels (n = 51, 80%), although non-bronchial systemic arteries were simultaneously embolized in 34 (53%) cases. The median number of vessels embolized per procedure was 3 (range 2–3). However, there were no differences between two groups in the type or number of vessels embolized.

**Table 2:** Angiographic findings and bronchial artery embolization in patients with pulmonary aspergillosis.

		All patients (N = 64)	CPA (n = 55)	SA (n = 9)	*P* value
Angiographic findings*					
	Hypervascularity	60 (94)	52 (95)	8 (89)	0.463
	Systemic-pulmonary shunt	48 (75)	42 (76)	6 (67)	0.679
	Extravasation	50 (78)	44 (80)	6 (67)	0.397
	Neovascularization	46 (72)	41 (75)	5 (56)	0.255
Embolization*					
	Bronchial artery	51 (80)	42 (76)	9 (100)	0.185
	Nonbronchial systemic artery	47 (73)	42 (76)	5 (56)	0.230
	Number of embolized artery	3 (2–3)	3 (2–3)	2 (1–3)	0.109

* Cases are duplicated.

The data are presented as median (interquartile range) or number (%).

CPA, chronic pulmonary aspergillosis; SA, simple aspergilloma

**Fig 1:**
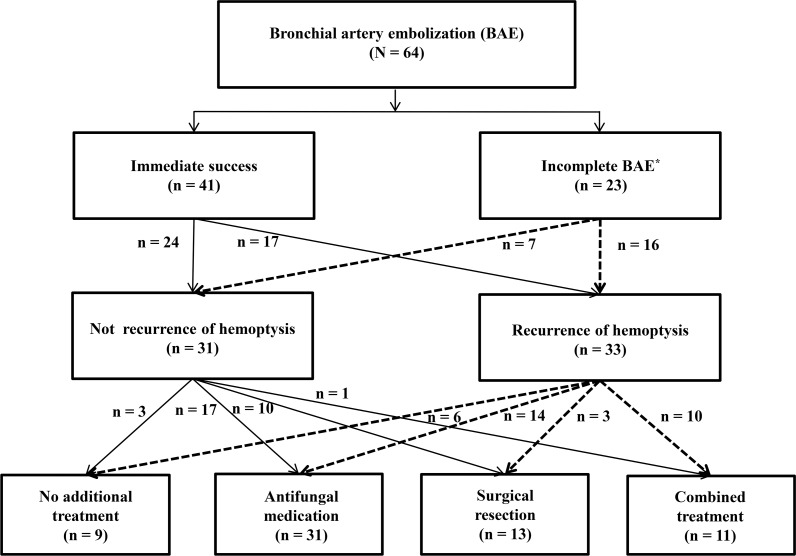
Flow chart of the study population with CPA who underwent BAE for life-threatening hemoptysis. CPA, chronic pulmonary aspergillosis; BAE, bronchial artery embolization. * One case of clinical failure of the first BAE is included.

The overall clinical outcomes of the patients who underwent BAE are summarized in Table 3. Immediate success was achieved with the first BAE in 41 (64%) patients. In the remaining 23 (36%) patients, the embolization could not be completed due to difficulty with the approach (n = 21) and/or overuse of contrast medium (n = 12). Clinical failure of the first BAE was observed in only one (2%) patient. Complications post-BAE were observed in four (6%) patients. During the median follow-up period of 26.0 (13.0–55.0) months, recurrence of hemoptysis was seen in a total of 33 patients (52%) at a median of 2.0 (0.3–10.0) months after the first BAE. Of these patients, repeat BAE was performed in 25 (25/33, 76%) patients. Additional treatments for pulmonary aspergillosis were necessary in 55 (86%) patients, which consisted of antifungal medication in 31 (48%) and surgical resection in 24 (38%) (Fig 1).

**Table 3:** Clinical outcomes of the patients with pulmonary aspergillosis underwent bronchial arterial embolization for life-threatening hemoptysis.

			All patients (N = 64)	CPA (n = 55)	SA (n = 9)	*P* value
Outcomes of the first BAE						
	Immediate success		41 (64)	35 (64)	6 (67)	> 0.999
	Incomplete BAE*		23 (36)	20 (36)	3 (33)	> 0.999
		Difficult to approach	21 (33)	18 (33)	3 (33)	
		Overuse of contrast medium	12 (19)	10 (18)	2 (22)	
	Failure		1 (2)	1 (2)	0	> 0.999
Complications of the first BAE			4 (6)	4 (7)	0	> 0.999
	Embolic events		1 (2)	1 (2)	0	
	Procedure related discomfort		3 (5)	3 (6)	0	
Recurrence of hemoptysis			33 (52)	30 (55)	3 (33)	0.296
	Time to recurrence, months		2.0 (0.3–10.0)	2.0 (0.3–10.0)	2.0 (0.5–NA)	0.916
		<1 month (early onset)	12 (19)	11 (20)	1 (11)	> 0.999
		1 month– 1 year	14 (22)	12 (22)	2 (22)	> 0.999
		>1 year	7 (11)	7 (13)	0	0.580
Repeated BAE after first BAE (n = 33)			25 (76)	22 (73)	3 (100)	0.560
Additional treatments for pulmonary aspergillosis						
	No additional treatment		9 (14)	8 (15)	1 (11)	> 0.999
	Antifungal medication		31 (48)	31 (56)	0	0.002
	Surgical resection		24 (38)	16 (29)	8 (89)	0.001
Mortality			15 (23)	15 (27)	0	0.101

* Cases are duplicated.

The data are presented as median (interquartile range) or number (%).

BAE, bronchial artery embolization; CPA, chronic pulmonary aspergillosis; SA, simple aspergilloma

**Fig 2:**
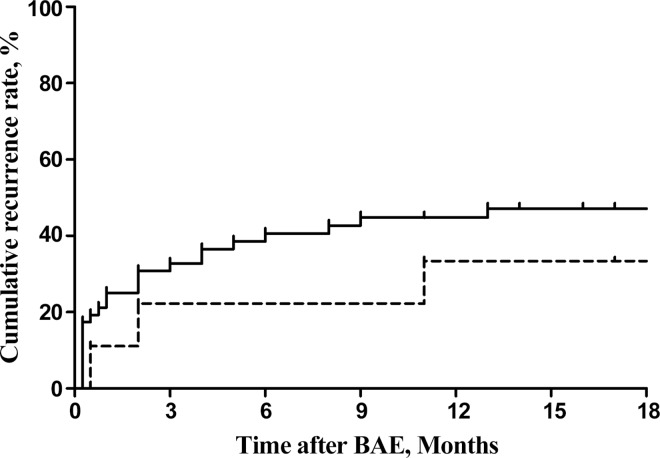
Cumulative recurrence rates following BAE in patients with CPA (solid line) and patients with SA (dotted line) (*P* = 0.061, log-rank test). BAE, bronchial artery embolization; CPA, chronic pulmonary aspergillosis.

In comparing the outcomes of patients with CPA and SA, there were no significant differences in rates of immediate success, incomplete embolization, or clinical failure of the first BAE (Table 3). The cumulative rates of recurrence following BAE in the two groups are shown in Fig 2. Recurrence of hemoptysis tended to be more common in patients with CPA (30, 55%) than in patients with SA (3, 33%) (*P* = 0.296).

In addition, antifungal medications following BAE were more commonly prescribed in the CPA group (56%) compared to the SA group (0%) (*P* = 0.002). All but one (89%) patient with SA required surgical resection after the first BAE, in contrast to 16 (29%) patients with CPA (*P* = 0.001). Finally, the overall mortality was 23%, which tended to be higher in patients with CPA (27%) compared to patients with SA (0%, *P* = 0.101).

## Discussion

To our knowledge, this is the first report of the clinical characteristics and outcomes of BAE in CPA patients with life-threatening hemoptysis. Our results demonstrated that BAE was a safe and effective procedure for the management of life-threatening hemoptysis due to CPA. However, recurrence of hemoptysis was common, and repeated BAE and antifungal treatment were required in the majority of patients. When comparing the complication rates of initial BAE, patients with SA and CPA did not show any differences in the rates of immediate success, incomplete embolization, or clinical failure. Despite this, the recurrence of hemoptysis tended to be higher in patients with CPA than in patients with SA.

Although there are many causes of hemoptysis,[24,25] life-threatening hemoptysis requiring intervention most often occurs in the setting of chronic inflammatory lung disease, predominantly in cases of pulmonary tuberculosis (TB) and bronchiectasis.[25] However, CPA, including SA, can also be complicated by mild, moderate, or life-threatening hemoptysis.[2] Although mild or moderate hemoptysis is frequently controlled with the use of conservative therapy, BAE has become a mainstay in the treatment of hemoptysis, especially in case of both life-threatening and recurring hemoptysis.[26] Numerous studies have demonstrated the effectiveness and success of BAE, especially in TB patients.[23,27–29] However, the recurrence of hemoptysis following successful BAE remains common and is associated with aspergilloma.[22,27,29] In the present study, the recurrence rate in patients with CPA was 55%, which is approximately two times higher than that of TB patients.[23,27–29] In addition, the majority of patients with recurrence of hemoptysis required repeat BAE. Therefore, additional treatments after a successful BAE are recommended to ensure the long-term success of the embolization in CPA patients.[1,4,30]

The chronic forms of pulmonary aspergillosis are SA, chronic cavitary pulmonary aspergillosis, and chronic necrotizing pulmonary aspergillosis,[1] although recent guidelines from the European Society for Clinical Microbiology and Infectious Diseases and the European Respiratory Society suggest new a classification of CPA to include aspergillus nodules and subacute invasive aspergillosis, which were previously termed chronic necrotizing pulmonary aspergillosis.[3] Although few data on BAE outcomes in patients with SA have been reported,[18–20] there have been no reports on clinical outcomes and complications of BAE in patients with other CPA subtypes. In this study, the outcome of BAE in patients with SA was consistent with previous reports.[18,20] All of the angiographic findings and embolization were similar in the SA and CPA subtype groups; however, hemoptysis recurrence tended to be higher in patients with other CPA subtypes. Recurrent hemoptysis within the first month of embolization is caused by incomplete embolization of the abnormal vessels,[26] which might be associated with the widespread tissue involvement found in other CPA subtypes. In addition, progression of CPA might cause recanalization of previously embolized vessels or revascularization of collateral circulation and result in late rebleeding following BAE.[22] For this reason, additional treatment including antifungal medication and surgical resection were required in the majority of the patients we studied.

Although this study provides new information on the clinical characteristics and outcomes of BAE in CPA patients, it is limited by its retrospective, observational design and the acquisition of data from a single center. Furthermore, there may have been insufficient statistical power to identify some significant findings due to the small sample size. Finally, the evaluation and therapeutic decision-making following BAE were not based on a pre-set protocol, but rather on the clinical experience of each attending physician. Therefore, the number of patients having antifungal medications before embolization was low.

## Conclusions

In conclusion, BAE was a safe and effective procedure for the management of life-threatening hemoptysis in patients with pulmonary aspergillosis. However, recurrence of hemoptysis was common, especially in patients with CPA. Therefore, definitive treatment for CPA following successful BAE should be considered to ensure the long-term success of the embolization in these patients.

## Abbreviations

BAE: bronchial artery embolization
CPA: chronic pulmonary aspergillosis
CT: computed tomography
IQR: interquartile range
SA: simple aspergilloma
TB: pulmonary tuberculosis
